# Comparison of Six Chromogenic Agar Media for the Isolation of a Broad Variety of Non-O157 Shigatoxin-Producing *Escherichia coli* (STEC) Serogroups

**DOI:** 10.3390/ijerph120606965

**Published:** 2015-06-17

**Authors:** Bavo Verhaegen, Koen De Reu, Marc Heyndrickx, Lieven De Zutter

**Affiliations:** 1 Institute for Agricultural and Fisheries Research (ILVO), Technology and Food Science Unit, Brusselsesteenweg 370, 9090 Melle, Belgium; E-Mails: Bavo.Verhaegen@ilvo.vlaanderen.be (B.V.); Marc.Heyndrickx@ilvo.vlaanderen.be (M.H.); 2 Faculty of Veterinary Medicine, Department of Veterinary Public Health and Food Safety, Ghent University, Salisburylaan 133, 9820 Merelbeke, Belgium; E-Mail: lieven.dezutter@ugent.be; 3 Department of Pathology, Bacteriology and Poultry Diseases, Faculty of Veterinary Medicine, Ghent University, Salisburylaan 133, 9820 Merelbeke, Belgium

**Keywords:** shigatoxin-producing *E. coli*, non-O157, chromogenic, isolation, agar media, quantitative

## Abstract

The isolation of non-O157 STEC from food samples has proved to be challenging. The selection of a suitable selective isolation agar remains problematic. The purpose of this study was to qualitatively and quantitatively evaluate six chromogenic agar media for the isolation of STEC: Tryptone Bile X-glucuronide agar (TBX), Rainbow^®^ Agar O157 (RB), Rapid *E. coli* O157:H7 (RE), Modified MacConkey Agar (mMac), CHROMagar^TM^ STEC (Chr ST) and chromID^TM^ EHEC (Chr ID). During this study, 45 *E. coli* strains were used, including 39 STEC strains belonging to 16 different O serogroups and 6 non-STEC *E. coli*. All *E. coli* strains were able to grow on TBX and RB, whereas one STEC strain was unable to grow on Chr ID and a number of other STEC strains did not grow on mMac, CHROMagar STEC and Rapid *E. coli* O157:H7. However, only the latter three agars were selective enough to completely inhibit the growth of the non-STEC *E. coli*. Our conclusion was that paired use of a more selective agar such as CHROMagar STEC together with a less selective agar like TBX or Chr ID might be the best solution for isolating non-O157 STEC from food.

## 1. Introduction

The multitude of infectious diseases transmitted by microorganisms is a burden for public health. The well-known shigatoxin-producing *Escherichia coli* (STEC), also known as verotoxin-producing *E. coli* (VTEC), causes human infections through direct transmission from person to person or from infected animals. It can also be indirectly transmitted via contaminated food, water, or environments contaminated with faeces [1]. STEC infections can be responsible for clinical symptoms ranging from mild to severe diarrhea, possibly complicated with hemolytic uremic syndrome (HUS) or thrombotic thrombocytopaenic purpura (TTP) [2]. Rapid detection of this pathogen is of utmost importance to ensure appropriate actions to safeguard public health. The recently increased use of highly-automated real-time PCR screening techniques provides the required highly-sensitive detection of all STEC. However, the follow-up culture–based isolation of the pathogen can be labour-intensive and time-consuming due to the long incubation period. In some cases such isolation is even unsuccessful due to lack of sufficiently selective isolation media [3]. Because, STEC O157:H7 was initially the most common serotype within the STEC group, the development of isolation media has been targeted for this serotype. The current cultural method of STEC O157:H7 is based on its inability to ferment sorbitol, its lack of β-D-glucuronidase enzyme activity and its resistance to selective agents such as potassium tellurite, novobiocin and cefixime [4]. Consequently, multiple selective isolation media with chromogenic substrates have been formulated for the isolation of O157:H7 [5]. These isolation media fail to detect atypical O157 STEC in addition to a large number of non-O157 STEC strains. However, these strains are increasingly recognised and reported as important foodborne pathogens worldwide; an important example is the STEC O104:H4 outbreak in Germany and France of 2011. In Europe the most frequently isolated and human pathogenic most important non-O157 STEC serogroups are O26, O91, O103, O111 and O145 [1,6,7]. In contrast to O157:H7, strains of these serogroups exhibit a broad variety of biochemical characteristics and a different sensitivity to selective agents. No single chromogenic isolation medium has yet been developed that allows cultivation and differentiation of all STEC from food samples [8]. Nevertheless, obtaining a verified positive isolate is crucial to confirm the positive results of the PCR-based screening techniques. In addition, culturing makes it possible to type the isolate in order to establish possible contamination routes and reveal important virulence factors [9].

Currently, the International Organization for Standardization (ISO) states that all samples in which a *stx* gene has been detected by PCR after enrichment should be further investigated by an isolation step on a selective agar medium. The use of Tryptone Bile X-glucuronide agar (TBX) for isolation of STEC is recommended. Since this medium lacks selectivity for STEC multiple presumptive positive colonies (up to 50) are routinely confirmed, and the choice of another medium is therefore allowed [10]. For this purpose a number of agar media has been developed; they can be either specific for isolation of *E. coli* O157, O26, or for all STEC in general. The growth capabilities and morphologies of many STEC serotypes on these selective media have not been thoroughly investigated, however.

The aim of this study was to evaluate the growth capacity and colony colours of a broad variety of STEC serotypes on several chromogenic media used for the isolation of *E. coli* O157 and other STEC, regardless of the O serogroup. The features of some non-STEC *E. coli* were also investigated on those media as a comparison [11].

## 2. Experimental Section

### 2.1. Strains

Table 1 lists the 45 *E. coli* strains used in this study. A total of 39 STEC belonging to the four most common and 12 less common non-O157 STEC serogroups were examined. Most strains were isolated from human patients by the Belgian national reference laboratory (Prof. Dr. Denis Piérard, UZ Brussels, Belgium); others originated from Belgian food samples. In addition, six non-STEC *E. coli* were included: two enteropathogenic *E. coli* (EPEC) and four commensal *E. coli* all isolated from cattle faeces. All strains were stored at −80 °C using Pro-Lab Microbank cryovials (Pro-Lab, Vaughan, ON, Canada) according to the manufacturer’s instructions. Presence of *stx1*, *stx2*, *eae* and *hlyA* genes was analysed according to Botteldoorn *et al.* [12]. For the *ter B* gene presence the method described by Taylor *et al.* [13] was used. The strains were cultured onto Tryptone Soy Agar (TSA; Oxoid, Ltd., Basingstroke, UK) at 37 °C for 24 h. A single colony from these culture plates was transferred into Tryptone Soy Broth (TSB; Oxoid) and incubated at 37 °C for 24 h.

**Table 1:** Overview of STEC, EPEC and commensal *E. coli* strains.

Strains	Serotypes	Origin	Virulence Genes				
			*Stx 1*	*Stx 2*	*Eae*	*Hly A*	*Ter B*
MB 5323	O5:H-	human	+	−	−	+	−
MB 5324	O5:H-	human	+	−	+	+	−
MB 5321	O8:H-	human	−	+	−	−	−
MB 5322	O8:H9	human	−	+	−	−	−
MB 5325	O55:H12	human	+	−	−	−	−
MB 5312	O55:H7	human	−	+	+	−	−
MB 5342	O63:H6	human	−	+	+	+	−
MB 5313	O63:H6	human	−	+	+	−	−
MB 5334	O84:H-	human	−	+	+	+	−
MB 5333	O84:H28	human	+	−	+	+	+
MB 5336	O91:H-	human	−	+	−	+	−
MB 5335	O91:H21	human	−	+	−	+	−
MB 5339	O113:H2	human	−	+	+	−	−
MB 5338	O113:H21	human	−	+	−	−	+
MB 5950	O118:H16	human	+	−	+	+	+
MB 5951	O118:H16	human	+	−	+	−	+
MB 5337	O121:H19	human	−	+	+	+	+
MB 5326	O128:H-	human	+	+	−	+	−
MB 5327	O128:H-	human	−	+	+	−	−
MB 5329	O146:H28	human	−	+	−	−	+
MB 5328	O146:H-	human	+	+	−	+	+
MB 5340	O182:H34	human	+	−	+	+	+
MB 5341	O182:H25	human	+	−	+	+	+
MB 5948	O26:H11	Swab *	−	+	+	+	+
MB 5316	O26:H11	Milk *	+	−	+	+	+
MB 2658	O26:H11	human	+	−	+	+	+
MB 2775	O26:H11	human	−	+	+	+	+
MB 5307	O103:H2	swab	+	−	+	+	−
MB 5308	O103:H2	milk	+	−	+	+	−
MB 2654	O103:H2	human	+	−	+	+	−
MB 2651	O103:H2	human	+	−	+	+	−
MB 5949	O111:H2	swab	−	+	+	+	+
MB 5310	O111:H8	swab	+	+	+	+	+
MB 2679	O111:H-	human	+	−	+	+	+
MB 2654	O111:H-	human	+	+	+	+	+
MB 5305	O145:H28	swab	+	−	+	+	+
MB 5850	O145:H28	swab	−	+	+	+	+
MB 2655	O145:H-	human	+	−	+	+	+
MB 2820	O145:H-	human	+	+	+	+	+
MB 5952		cattle faeces	−	−	+		+
MB 5953		cattle faeces	−	−	+		+
MB 5956		cattle faeces	−	−	−		−
MB 5957		cattle faeces	−	−	−		−
MB 5958		cattle faeces	−	−	−		−
MB 5959		cattle faeces	−	−	−		−

* Swab: cattle carcass swab; milk: cow milk.

### 2.2. Selective Isolation Media

The following selective isolation media were evaluated. Tryptone Bile X-glucuronide agar (TBX; Bio-Rad, Marnes-la-Coquettes, France); Rainbow^®^ Agar O157 (RBA; Biolog Inc., Hayward, CA, USA) without supplementations; Rapid *E. coli* O157:H7 (RE; Bio-Rad), supplemented with 10 mg/L novobiocin and 0.8 mg/L potassium tellurite; Modified MacConkey Agar (mMac) for the isolation of non-O157 STEC strains as described by Possé *et al.* [14]. Briefly, this medium contains MacConkey agar base (BD Biosciences, Franklin Lakes, NJ, USA) supplemented with two sugars (sucrose and sorbose) and several selective components: 3.5 g/L bile salts No. 3 (Sigma Aldrich/Fluka, St-Louis, MO, USA), 0.05 g/L 5-bromo-4-chloro-3-indolyl β-D-galactopyranoside (X-gal, Glycosynth, Warrington, UK), 0.05 g/L isopropyl-β-D-thiogalactopyranoside (IPTG, Glycosynth), 8.0 mg/L novobiocin (Sigma) and 2.5 mg/L potassium tellurite (Sigma); CHROMagar STEC^TM^ supplemented with 10 mL/L selective mix (Chr ST; CHROMagar Microbiology, Paris, France) and the recently launched ChromID EHEC supplemented with 4 mL/L cefixime-tellurite mix (Chr ID; bioMérieux, Paris, France) were included.

### 2.3. Qualitative Study

After incubation 10 µL of each TSB strain culture was inoculated onto the six chromogenic agar media. All agar media were incubated at 37 °C for 24 h and visually examined for growth and colony morphology.

### 2.4. Quantitative Study

To determine the possible inhibition of growth of the STEC strains on the chromogenic agar media, all cultures grown in TSB were serially diluted in Peptone Water (Bio-Rad, Marnes-la-Coquettes, France) to a concentration of 10^4^ cfu/mL. One millilitre of each dilution was manually spread plated on two agar plates (each 0.5 mL) of each of the six agar media and TSA medium as reference. In addition 100 µL of each dilution was inoculated on one agar plate of each of the seven agar media using a spiral plate machine (Eddy Jet Spiral Plater, IUL Instruments, Barcelona, Spain). The plates were incubated for 24 h at 37 °C and the colonies counted. In accordance with Gill *et al.* [15], the efficiency of recovery was calculated as the percentage of counted colonies on the different selective isolation media compared to the enumeration obtained on the non-selective TSA. The study was replicated three-fold; the mean recovery percentage and standard error were calculated.

## 3. Results and Discussion

The isolation of non-O157 STEC strains from food samples has proved challenging due to the lack of known differential biochemically characteristics and inherent sensitivities to additives. For this reason the discrimination from other *E. coli* and other non-target organisms remains problematic [8,14]. Before the interference of an extensive background microbiota can be evaluated, the growth capabilities of STEC strains themselves should be investigated. In this study the growth of STEC on six chromogenic agar media was evaluated using a range of STEC serotypes often isolated in Belgium. Furthermore, the growth and appearance of these STEC strains was compared to some common non-STEC *E. coli*. In the threefold replicated experiment some natural variation in counts was observed, but the colony colours of the different cultures of STEC strains, based upon the enzymatic cleaving of chromogenic substrates and carbohydrate fermentation, remained the same.

### 3.1. Tryptone Bile X-Glucuronide Agar (TBX)

**Figure 1:**
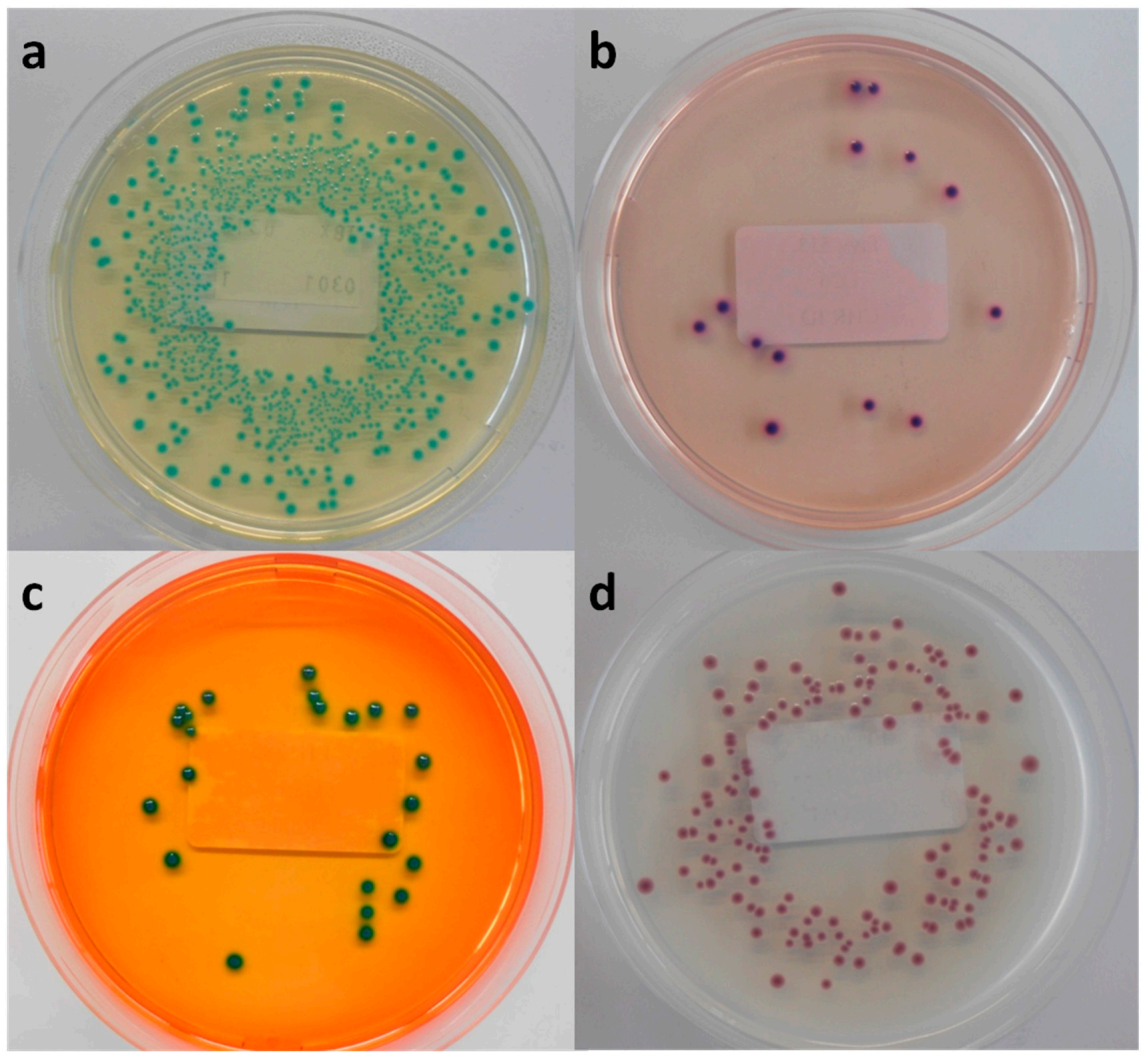
Macroscopic view of (**a**) Tryptone Bile X-glucuronide agar (TBX), (**b)** chromID EHEC agar (Chr ID), (**c**) Rapid *E. coli* O157:H7 agar (RE) and (**d**) CHROMagar STEC (Chr ST), inoculated with MB 5316 (**a**), MB 5948 (**b**), MB 5322 (**c**) and MB 5341 (**d**).

Among the six evaluated chromogenic media, TBX is designed to detect all *E. coli*, including STEC. Therefore, all tested STEC and non-STEC *E. coli* strains were able to grow on TBX (Table 2) and demonstrated the typical blue-green colour indicating the presence of β-glucuronidase activity Figure 1a. Moreover, the recovery percentage of the STEC serogroups and other non-STEC *E. coli* compared to TSA was at least 78% and 93%, respectively (Table 3), with averages for both groups of 101% and 97%, respectively. This ISO/TS 13136:2012 recommended agar medium contains selective agents inhibiting the growth of Gram-positive organisms and swarming by *Proteus* sp. [16]. It remains a valuable isolation medium for STEC, despite the labour-intensity and time-consuming practice of isolating multiple presumptive positive colonies (up to 50), pooling and confirming as described in the ISO/TS, due to its low selectivity.

**Table 2:** Growth and colony appearance of STEC and non-STEC *E. coli* strains on six chromogenic isolation media.

Strain	Serotype	*TerB*	TBX	RBA	RE	mMac	Chr ST	Chr ID
MB 5323	O5:H-	−	blue-green	grey-green	green	− *	−	purple
MB 5324	O5:H-	+	blue-green	grey-green	green	green	mauve	purple
MB 5321	O8:H-	−	blue-green	red	green	−	−	purple
MB 5322	O8:H9	−	blue-green	purple	green	−	−	−
MB 5325	O55:H12	+	blue-green	purple	green	grey-green	mauve	blue
MB 5312	O55:H7	−	blue-green	red	green	−	−	purple
MB 5342	O63:H6	−	blue-green	red	dark blue	−	−	purple
MB 5313	O63:H6	−	blue-green	red	dark blue	−	−	purple
MB 5334	O84:H-	+	blue-green	cream-white	yellow	yellow	mauve	white
MB 5333	O84:H28	+	blue-green	red-purple	green	green	mauve	purple
MB 5336	O91:H-	−	blue-green	purple	green	−	−	purple
MB 5335	O91:H21	−	blue-green	purple	green	−	−	purple
MB 5339	O113:H2	−	blue-green	red-purple	green	−	−	purple
MB 5338	O113:H21	+	blue-green	red-purple	green	grey-green	mauve	purple
MB 5950	O118:H16	+	blue-green	purple	green	red	mauve	purple
MB 5951	O118:H16	+	blue-green	purple	green	grey-green	mauve	purple
MB 5337	O121:H19	+	blue-green	red	yellow	red	mauve	red
MB 5326	O128:H-	−	blue-green	red-purple	green	−	−	purple
MB 5327	O128:H-	−	blue-green	red-purple	green	−	−	purple
MB 5329	O146:H28	+	blue-green	purple	green	green	mauve	purple
MB 5328	O146:H-	+	blue-green	purple	green	grey-green	mauve	purple
MB 5340	O182:H34	+	blue-green	purple	dark blue	green	mauve	purple
MB 5341	O182:H25	+	blue-green	purple	dark blue	green	mauve	purple
MB 5948	O26:H11	+	blue-green	purple	green	red	mauve	purple
MB 5316	O26:H11	+	blue-green	purple	green	red	mauve	purple
MB 2658	O26:H11	+	blue-green	purple	green	red	mauve	purple
MB 2775	O26:H11	+	blue-green	purple	green	red	mauve	green
MB 5307	O103:H2	−	blue-green	purple	green	blue-green	mauve	purple
MB 5308	O103:H2	−	blue-green	purple	green	blue-green	mauve	purple
MB 2654	O103:H2	−	blue-green	purple	green	−	−	purple
MB 2651	O103:H2	−	blue-green	purple	green	−	−	purple
MB 5949	O111:H2	+	blue-green	grey-green	green	grey-green	mauve	purple
MB 5310	O111:H8	+	blue-green	grey-green	green	grey-green	mauve	purple
MB 2679	O111:H-	+	blue-green	grey-green	green	grey-green	mauve	purple
MB 2654	O111:H-	+	blue-green	grey-green	green	grey-green	mauve	purple
MB 5305	O145:H28	+	blue-green	purple	green	green	mauve	purple
MB 5850	O145:H28	+	blue-green	purple	green	green	mauve	purple
MB 2655	O145:H-	+	blue-green	purple	green	green	mauve	purple
MB 2820	O145:H-	+	blue-green	purple	green	green	mauve	purple
MB 5952		+	blue-green	purple	green	green	mauve	purple
MB 5953		+	blue-green	purple	green	green	mauve	green
MB 5956		−	blue-green	purple	−	−	−	red
MB 5957		−	blue-green	purple	−	−	−	purple
MB 5958		−	blue-green	purple	−	−	−	purple
MB 5959		−	blue-green	purple	green	−	−	purple

TBX: Tryptone Bile X-glucuronide agar, RBA: Rainbow^®^ Agar O157, RE: Rapid *E. coli* O157:H7, mMac: Modified MacConkey Agar as described by Possé *et al.* [14], Chr ST: CHROMagar STEC^TM^, Chr ID: Chrom ID EHEC, −: no growth.

**Table 3:** The mean recovery percentages of the STEC and non-STEC strains ± standard error on the six chromogenic isolation media compared with the growth on TSA medium (the average concentration of the inocula based on the logarithmic counts on Tryptone Soy Agar (TSA): 4.13 ± 0.12 log_10_ cfu/mL). TBX: Tryptone Bile X-glucuronide agar, RB: Rainbow^®^ Agar O157, RE: Rapid *E. coli* O157:H7, mMac: Modified MacConkey Agar as described by Possé *et al.* [14], Chr ST: CHROMagar STEC, Chr ID: ChromID EHEC.

Strain	Serotype	*TerB*	TBX	RB	RE	mMac	Chr ST	Chr ID
								
MB 5323	O5:H-	−	116 ± 15	114 ± 10	− *	−	−	<1
MB 5324	O5:H-	+	91 ± 10	104 ± 9	−	60 ± 9	60 ± 18	85 ± 26
MB 5321	O8:H-	−	94 ± 4	95 ± 10	−	−	−	41 ± 13
MB 5322	O8:H9	−	97 ± 8	109 ± 15	2 ± 1	−	−	−
MB 5325	O55:H12	+	84 ± 8	119 ± 15	−	51 ± 18	34 ± 7	42 ± 18
MB 5312	O55:H7	−	110 ± 16	114 ± 11	−	−	−	30 ± 4
MB 5342	O63:H6	−	90 ± 18	68 ± 10	−	−	−	33 ± 10
MB 5313	O63:H6	−	92 ± 8	90 ± 18	−	−	−	78 ± 25
MB 5334	O84:H-	+	107 ± 11	89 ± 15	−	101 ± 21	108 ± 14	107 ± 36
MB 5333	O84:H28	+	96 ± 6	106 ± 9	−	86 ± 33	64 ± 7	68 ± 21
MB 5336	O91:H-	−	82 ± 15	108 ± 2	−	−	−	<1
MB 5335	O91:H21	−	95 ± 32	99 ± 9	−	−	−	34 ± 7
MB 5339	O113:H2	−	115 ± 12	108 ± 4	30 ± 8	−	−	26 ± 7
MB 5338	O113:H21	+	78 ± 23	108 ± 5	−	29 ± 8	51 ± 12	38 ± 10
MB 5950	O118:H16	+	95 ± 5	101 ± 20	−	36 ± 6	57 ± 10	32 ± 7
MB 5951	O118:H16	+	104 ± 18	116 ± 5	−	47 ± 14	101 ± 15	54 ± 8
MB 5337	O121:H19	+	96 ± 1	93 ± 5	−	142 ± 49	122 ± 12	72 ± 9
MB 5326	O128:H-	−	88 ± 12	36 ± 9	−	−	−	26 ± 6
MB 5327	O128:H-	−	84 ± 31	81 ± 11	−	−	−	<1
MB 5329	O146:H28	+	94 ± 15	93 ± 10	−	6 ± 6	33 ± 9	33 ± 9
MB 5328	O146:H-	+	125 ± 20	94 ± 7	−	26 ± 1	6 ± 1	26 ± 4
MB 5340	O182:H34	+	110 ± 12	88 ± 4	−	44 ± 2	33 ± 4	85 ± 19
MB 5341	O182:H25	+	100 ± 3	116 ± 12	−	33 ± 8	30 ± 10	18 ± 3
MB 5948	O26:H11	+	83 ± 15	107 ± 2	−	<1	60 ± 19	<1
MB 5316	O26:H11	+	89 ± 20	88 ± 6	−	100 ± 24	82 ± 16	15 ± 5
MB 2658	O26:H11	+	99 ± 6	96 ± 17	−	<1	48 ± 16	6 ± 0
MB 2775	O26:H11	+	116 ± 6	88 ± 11	2 ± 1	2 ± 0	35 ± 6	29 ± 2
MB 5307	O103:H2	−	113 ± 9	106 ± 7	−	−	−	73 ± 12
MB 5308	O103:H2	−	109 ± 7	112 ± 5	−	−	−	63 ± 7
MB 2654	O103:H2	−	119 ± 17	105 ± 11	−	−	−	98 ± 5
MB 2651	O103:H2	−	93 ± 0	110 ± 2	−	−	−	79 ± 13
MB 5949	O111:H2	+	114 ± 8	113 ± 1	−	29 ± 9	24 ± 4	50 ± 6
MB 5310	O111:H8	+	110 ± 14	98 ± 5	−	23 ± 3	47 ± 7	49 ± 15
MB 2679	O111:H-	+	122 ± 8	113 ± 5	−	43 ± 6	59 ± 15	44 ± 15
MB 2654	O111:H-	+	100 ± 2	100 ± 14	−	39 ± 11	65 ± 7	71 ± 4
MB 5305	O145:H28	+	120 ± 17	121 ± 16	−	49 ± 13	40 ± 10	26 ± 7
MB 5850	O145:H28	+	96 ± 10	87 ± 10	−	42 ± 10	72 ± 22	4 ± 1
MB 2655	O145:H-	+	112 ± 13	96 ± 15	−	67 ± 13	23 ± 5	25 ± 6
MB 2820	O145:H-	+	100 ± 8	102 ± 23	−	51 ± 7	24 ± 5	4 ± 0
MB 5952		+	96 ± 13	111 ± 9	7 ± 3	56 ± 11	60 ± 18	72 ± 21
MB 5953		+	116 ± 12	110 ± 21	17 ± 9	29 ± 21	66 ± 11	50 ± 10
MB 5956		−	96 ± 13	102 ± 19	−	−	−	11 ± 9
MB 5957		−	116 ± 12	118 ± 36	−	−	−	15 ± 14
MB 5958		−	98 ± 19	118 ± 21	−	−	−	4 ± 2
MB 5959		−	93 ± 13	72 ± 17	2 ± 1	−	−	1 ± 1

* −: no growth (<1 cfu/mL).

### 3.2. Rainbow^®^ Agar O157(RBA)

**Figure 2:**
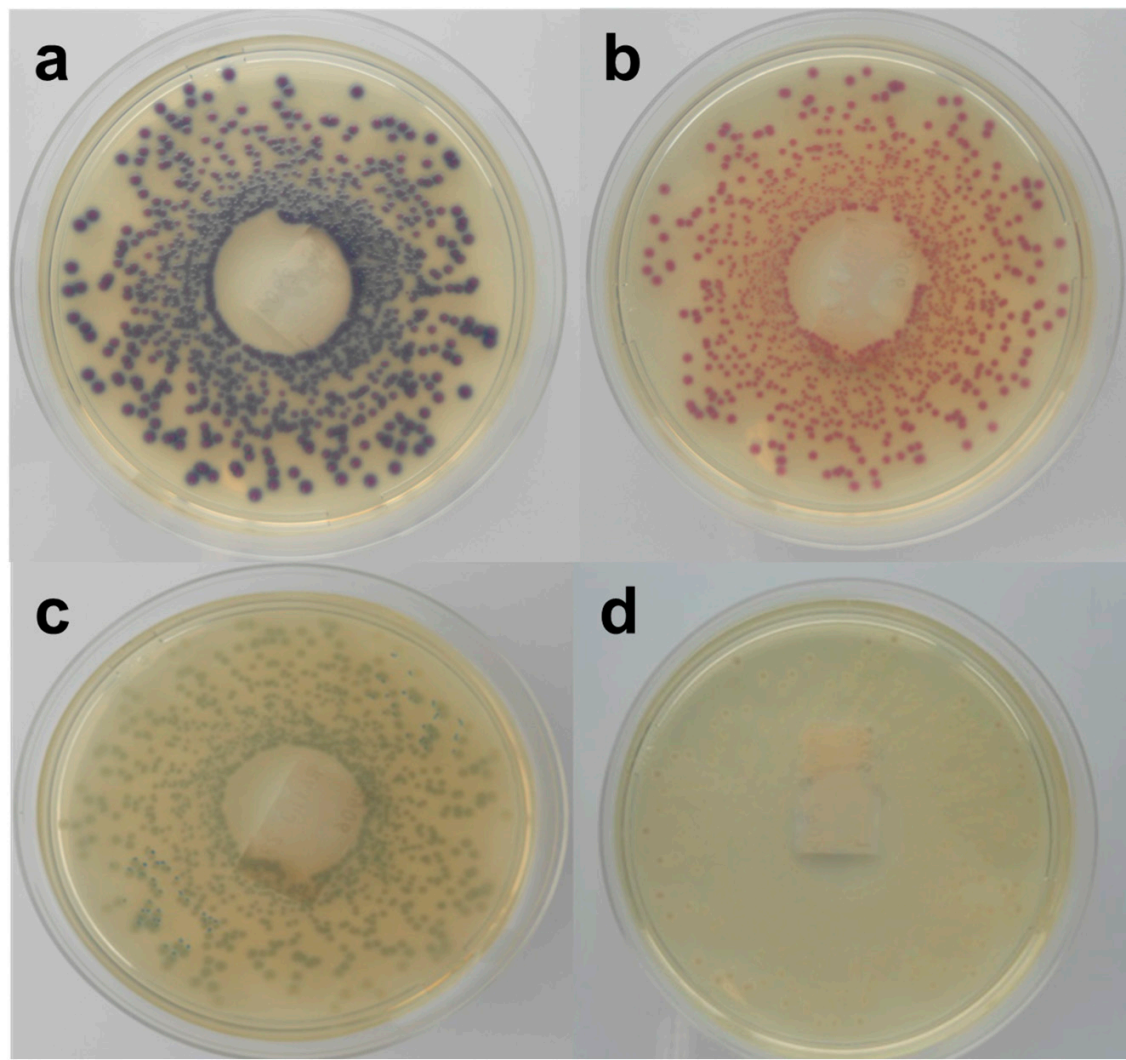
Macroscopic view of Rainbow O157 agar inoculated with STEC strains, (**a**) purple colonies (MB 5322), (**b**) red colonies (MB 5313), (**c**) grey-green colonies (MB 5324) and (**d**) cream-white colonies (MB 5334).

In accordance with TBX, RBA was able to support the growth of all tested STEC and non-STEC *E. coli* strains, and the average recovery percentage of the STEC serogroups and other non-STEC *E.* *coli* was similar this for TSA (approximately 100%). The colony colours of the different strains on RB ranged from purple–red–pink-grey-green-to cream-white Figure 2. Remarkably, the colonies of strains belonging to the same O-serogroup were not always consistent in color. Non-STEC *E. coli* strains could not be differentiated from STEC strains based on colony colours.

Modifications to this RBA base have been described to support growth of STEC while allowing more selectivity towards non-target organisms. Supplementation of the medium with 0.8 mg/L potassium tellurite and 10 mg/L sodium novobiocin is recommended by the manufacturer for samples with high microbiological background, whereas 0.05 mg/L cefixime, 0.15 mg/L potassium tellurite, and 5 mg/L novobiocin is recommended by the USDA STEC [17]. However, multiple studies demonstrated that both supplementations were unable to support the growth of a substantial proportion of STEC strains tested [9,15,18]. Further, Kase *et al.* [9] showed that the addition of washed sheep’s blood to RBA substantially reduced these inhibitions. However, in the present study only the Rainbow agar base without any supplementations was evaluated. The observed broad variety of colony colours and the inability to distinguish between STEC and non-STEC *E. coli* was considered problematic to select colonies for confirmation testing.

### 3.3. Rapid E. coli O157:H7 (RE)

In the qualitative study RE supported the growth of all tested STEC strains and half of the non-STEC *E. coli* strains. All colonies showed a green morphology (Figure 1c), except for two STEC serogroups (O63, O182), which presented a characteristic dark blue colour typical for *E. coli* O157:H7. When RE was inoculated with lower concentrated inocula only three STEC and three non-STEC *E.* *coli* strains were able to form colonies, with a very low average recovery percentage of 11% and 9%, respectively. In general, RE showed a significant selectivity towards STEC in both colony morphology and growth inhibition, making this agar medium unsuitable for isolation of non-O157 STEC strains.

### 3.4. CHROMagar^TM^ STEC (Chr ST)

In the present study only 24 STEC strains (61%) were able to develop colonies after inoculation at the low dose on Chr ST. The STEC and non-STEC *E. coli* strains that were able to grow presented colonies in many shades of mauve and often displayed different edges (Figure 1d). Moreover, the average recovery percentage was 53% and 63% compared to TSA, respectively. This high inhibition has already been observed in multiple studies carried out since the launch of Chr ST [9,15,19,20,21]. Both Hirvonen *et al.* [19] and Tzschoppe *et al.* [20] remarked on strong association between the growth on Chr ST and the presence of *terD* and *terB* of the *Ter* gene cluster, respectively. This gene complex contains four essential genes (*TerB*, *TerC*, *TerD* and *TerE*) conferring the resistance to strong oxidizing agent tellurite. These tellurite-resistant bacteria reduce tellurite to its less toxic form, which accumulates as black pigment inside the cell [22,23,24]. In the present study *TerB* was selected as marker for the *Ter* gene cluster. Twenty-four of the tested STEC strains were *terB*-positive and all were able to grow on Chr ST. Furthermore, all non-STEC *E. coli* strains failed to develop colonies, except the two *terB*-positive EPEC strains. This finding confirms the strong association between the growth on Chr ST and the presence of *terB*. Moreover, it was observed that only a small proportion of the *eae*-negative STEC strains were able to grow [9,15,19,20]. In the present study 10 *eae*-negative strains were included; only four grew on Chr ST.

### 3.5. Modified MacConkey Agar (mMac)

**Figure 3:**
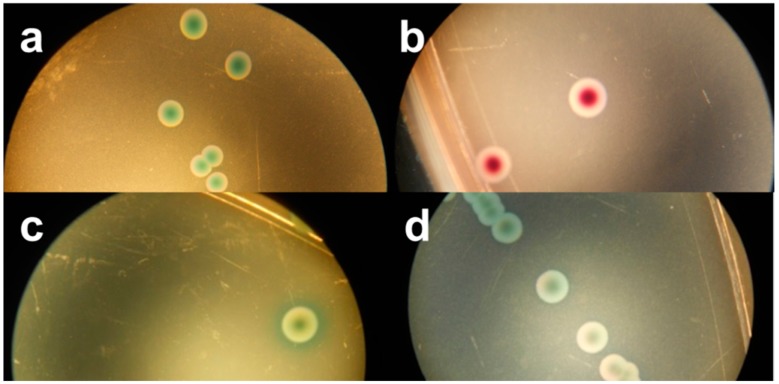
Stereo-microscopic view (**a**–**d**) of modified MacConkey agar as described by Possé *et al.* [14], inoculated with STEC strains. ((**a**) STEC O145 (MB 5850), (**b**) STEC O26 (MB 5316), (**c**) STEC O103 (MB 5307) and (**d**) STEC O111 (MB 2679)).

mMac was originally designed to differentiate between the four most common non-O157 STEC serogroups (O26, O103, O111, O145), using the colony colours dependent on the β-D-galactosidase activity and carbohydrate fermentation of these four serogroups (Figure 3). While the tested STEC strains belonging to this serogroup presented the predicted colony colours, the growth of two O103 strains was not supported. Most of the other serogroups showed similar colours, while some exhibited atypical colours (yellow). In accordance with RBA, the colonies of strains of the same O-serogroup were not always consistent in colour. Moreover, the color differences found in our study were often subtle and hard to discriminate. Still, Verstraete *et al.* [25] indicated its effectiveness as isolation medium for non-O157 STEC in food during the validation by an international ring trial. Nevertheless, the same STEC and non-STEC *E. coli* strains that failed to develop colonies on Chr ST also failed on mMac. The STEC that were able to grow showed an average recovery percentage of 50% compared to TSA. On the other hand, all non-STEC *E. coli* strains failed to develop colonies, except the two EPEC strains whose morphology was similar to the O145 STEC strains. Similar to Chr ST, a correlation between the growth on mMac and the presence of *terB* was observed.

### 3.6. ChromID EHEC (Chr ID)

The novel isolation agar chromID EHEC supported the growth of all tested STEC and non-STEC *E. coli* strains, except for one STEC (O8:H9) and one non-STEC *E. coli* strain. However, the strains able to develop colonies showed significant reduced recovery percentages compared to TSA: 41% and 26%, respectively. The appearance on Chr ID did not distinguish STEC from non-STEC *E. coli* colonies-they were all purple (Figure 1b), with a few exceptions. Despite the supplementation of an unspecified cefixime-tellurite mix to Chr ID, no correlation could be observed between the presence of *terB* and the growth on this medium.

## 4. Conclusions

The observed strong inhibition of Rapid *E. coli* O157:H7 towards STEC strains other than O157 makes the agar medium unsuitable for STEC isolation. Due to the high level of selectivity observed for Chr ST and mMac, most non-target organisms were sufficiently inhibited on these agars, which may facilitate the isolation of the major part of the tested STEC strains. Still, the isolation is limited to those strains that show resistance to the selective ingredients supplemented (e.g., tellurite, cefixime) to the chromogenic media. On the other hand, the biochemical characteristics (*i.e*., fermentative profile) of the different STEC strains are too diverse to use chromogenic media without supplementations. Therefore, the sole use of one of these two agars for the isolation of STEC from food might result in false negative. Paired use of two tested agar media might therefore be a useful option. The practical experience in this study with the more selective but easier to read Chr ST, in combination with a less selective agar like Chr ID or TBX, which allows the growth of all STEC strains, might be the best solution at present. However, in this study the observed colony colours and growth using pure cultures could only indicate the suitability of the media to support growth of the different STEC serotypes. In the presence of background micro-organisms or other STEC serotypes, adjacent colonies could influence the colony colour and growth of the target organisms [8]. In future research the isolation capabilities of combining two agars will be investigated using more complex food environment.
